# Mapping, Infrastructure, and Data Analysis for the Brazilian Network of Rare Diseases: Protocol for the RARASnet Observational Cohort Study

**DOI:** 10.2196/24826

**Published:** 2021-01-22

**Authors:** Domingos Alves, Diego Bettiol Yamada, Filipe Andrade Bernardi, Isabelle Carvalho, Márcio Eloi Colombo Filho, Mariane Barros Neiva, Vinícius Costa Lima, Têmis Maria Félix

**Affiliations:** 1 Department of Social Medicine Ribeirão Preto Medical School University of São Paulo Ribeirão Preto Brazil; 2 Public Health Postgraduate Program Ribeirão Preto Medical School University of São Paulo Ribeirão Preto Brazil; 3 Bioengineering Postgraduate Program School of Engineering University of São Paulo São Carlos Brazil; 4 Ribeirão Preto Medical School University of São Paulo Ribeirão Preto Brazil; 5 Institute of Mathematics and Computer Sciences University of São Paulo São Carlos Brazil; 6 Medical Genetics Service Porto Alegre Clinical Hospital Porto Alegre Brazil

**Keywords:** rare disease, digital health, health observatory, data science, health network

## Abstract

**Background:**

A rare disease is a medical condition with low prevalence in the general population, but these can collectively affect up to 10% of the population. Thus, rare diseases have a significant impact on the health care system, and health professionals must be familiar with their diagnosis, management, and treatment.

**Objective:**

This paper aims to provide health indicators regarding the rare diseases in Brazil and to create a network of reference centers with health professionals from different regions of the country. RARASnet proposes to map, analyze, and communicate all the data regarding the infrastructure of the centers and the patients’ progress or needs. The focus of the proposed study is to provide all the technical infrastructure and analysis, following the World Health Organization and the Brazilian Ministry of Health guidelines.

**Methods:**

To build this digitized system, we will provide a security framework to assure the privacy and protection of each patient when collecting data. Systems development life cycle methodologies will also be applied to align software development, infrastructure operation, and quality assurance. After data collection of all information designed by the specialists, the computational analysis, modeling, and results will be communicated in scientific research papers and a digital health observatory.

**Results:**

The project has several activities, and it is in an initial stage. Initially, a survey was given to all health care centers to understand the technical aspects of each network member, such as the existence of computers, technical support staff, and digitized systems. In this survey, we detected that 59% (23/39) of participating health units have electronic medical records, while 41% (16/39) have paper records. Therefore, we will have different strategies to access the data from each center in the data collection phase. Later, we will standardize and analyze the clinical and epidemiological data and use these data to develop a national network for monitoring rare diseases and a digital health observatory to make the information available. The project had its financing approved in December 2019. Retrospective data collection started in October 2020, and we expect to finish in January 2021. During the third quarter of 2020, we enrolled 40 health institutions from all regions of Brazil.

**Conclusions:**

The nature of rare disease diagnosis is complex and diverse, and many problems will be faced in the evolution of the project. However, decisions based on data analysis are the best option for the improvement of the rare disease network in Brazil. The creation of RARASnet, along with all the digitized infrastructure, can improve the accessibility of information and standardization of rare diseases in the country.

**International Registered Report Identifier (IRRID):**

DERR1-10.2196/24826

## Introduction

### Background

A rare disease (RD) is a medical condition with low prevalence compared to diseases prevalent in the general population, but there is no consensus on its definition [1]. The European Union describes a RD as a disease that affects no more than 1 person in 2000 [2]. In the United States, the Rare Disease Act of 2002 considers a RD any condition that affects less than 200,000 people across the country, or 1 person in 1500 [3]. In Japan, a RD is considered to affect less than 50,000 people (ie, 1 in 2500 people) [4]. On the other hand, in Latin America, there is no consensus on the definition of RDs in terms of numbers. Each country has its own definition according to its public policies, decrees, the existence of adequate treatments, or the severity of the disease [5].

Although individually rare, RDs collectively affect up to 10% of the population. Thus, rare diseases have a significant impact on health, and health professionals must be familiar with their diagnosis, management, and treatment [6]. It is estimated that there are up to 8000 rare diseases identified, 80% of which are of genetic origin [7]. In Brazil, the Ministry of Health defines a rare disease using the criteria of the World Health Organization (WHO), that is, 1.3 cases per 2000 individuals [8]. In 2014, the Brazilian Policy of Comprehensive Care for People with Rare Diseases was established within the scope of the Unified Health System (Sistema Único de Saúde [SUS]) [9].

To overcome this informational barrier, the WHO recommends that research involving the study of the process dynamics of RDs at the national level be financed by public agencies. The formation of large multidisciplinary networks is part of a fundamental process to encourage the collaboration of medical specialists, referral centers, and patient groups [10]. Providing an infrastructure for new mechanisms promoting the translation of basic research into clinically important products is still a priority. One of the most important opportunities addressed by the WHO reinforces support for “networks of excellence that focus on research infrastructures; research, infrastructure, and implementation of guidelines for medical and psychosocial care; [and] methods to provide easy access to health care available to patients, regardless of where they live” [11].

The first full version of a report relating to the concept of a health regionalized network emerged after World War I due to the consequent need for changes in the social protection system, presenting better ways for health services organization. Almost one century later, the original purpose remains very similar, as many health systems around the world offer a specific organization of services to attend to their population. A need to establish a uniform system of clinical histories was declared a crucial reason for the better integration of different health service levels [12].

The proposal to integrate health networks gained momentum with the advent of health observatories. The emergence of these epidemiological monitoring centers is related to rapid changes in the health sector, including the need to monitor and assess the impact of health programs and public health policies, the advent of informational intelligence, digital health, and health knowledge management. The health observatories’ functions include locating, gathering, analyzing, synthesizing, and disseminating data on the health status of a population, in addition to establishing partnerships and contacts with other agencies involved in the health of that region [13].

Independent of which model is used, the majority of countries today have digital systems to manage their data according to their health network structures. According to WHO recommendations, although digital health interventions are not enough on their own, when combined with health professionals, they are vital tools to promote health quality [11]. While data are still the main asset in the current digital world, many institutions do not yet fully understand the need and advantages of sharing their data with other organizations [14].

Cross-institutional sharing of health data is a challenge because many institutions are unwilling to share data due to privacy concerns or the fear of giving other institutions competitive advantages and, at an operational level, because of mistrust of technical barriers (there is no common platform for sharing these complex and heterogeneous data). On the other hand, overcoming this challenge may lead to better clinical effectiveness and improved clinical research [15]. Even if these concerns can be beaten, there is no consensus about the exact technical infrastructure needed to support such an effort.

Studies have shown that there remain a set of meaningful hurdles to achieving the desired benefits of health care data exchange. For example, failing to secure the patient record has financial and legal consequences as well as the negative potential to impact patient care. Thus, possible repercussions of a breach are a discouragement to swapping data. To avoid ethical and legal consequences for institutions, anonymization and privacy must be ensured for sensitive data, making them available only to authorized persons. Further, data anonymity could help improve the research area by removing identifiable information and sharing only limited data [15,16].

Another significant barrier that health networks need to face is adequate technological infrastructure. In addition to the scarce and fragmented availability of data on RDs [17], several technical aspects are common, like a centralized data source, which represents a high-security risk due to the susceptibility for malicious attacks to a nonredundant authority. A secure channel to send data to other organizations is another feature that institutions must address to avoid unauthorized access [14].

Due to the complexity of data in the health domain, achieving full interoperability is a hard task. Heterogeneous structures and data diversity decrease the accuracy of analysis and reduce understanding of information. To face this issue, several entities have created standards for data exchange. However, there is no consensus on the most adequate ones [18]. In Brazil, the Ministry of Health Ordinance 2073 of August 31, 2011, regulates the use of interoperability and health information standards for health information systems within the scope of the SUS; at the municipal, state, and federal levels; and in private systems and the private health sector [19].

In this sense, for there to be semantic interoperability between independent systems, it is necessary to standardize two aspects of information: the structure of the information and the semantic representation of the information. The information structure concerns the information and knowledge models that allow the systems to exchange data, formed in larger structures such as documents, correctly. The semantic representation of information includes terminologies, ontologies, and controlled vocabularies [20].

This framework can mitigate the several barriers preventing data access for research support agencies, academics, managers, and health professionals, such as the noncomputerization of processes, heterogeneity and duplicity of data in health information systems, and existence of a large amount of isolated data in databases accessible only in a certain context, usually to answer specific questions in particular research [17].

These factors often cause problems in the quality of information, making it difficult to coordinate and evaluate data in a research network linked to a rare disease patient care network, so it is not possible to use the data to assist in the decision-making process. Therefore, in health care, decision support tools are essential to guide the practice of health care and support the decisions of managers who will directly influence the quality of care provided to patients with RDs [21].

This paper presents a subproject belonging to the main project, entitled the Brazilian Rare Disease Network, funded by the Brazilian Council for Scientific and Technological Development (Conselho Nacional de Desenvolvimento Científico e Tecnológico [CNPq]), with a 2-year forecast. The main project is a mixed prospective and retrospective observational cohort study to map the landscape of rare diseases in Brazil. RARASnet is responsible for infrastructure and data analysis to provide health indicators and support the construction, organization, and monitoring of this patient network. Although the objectives are collaborative, different teams are responsible for specific parts.

### Related Work

RDs represent a major challenge for the organization of health care. The cooperation of health service professionals, civil society, and academia is essential to overcome this challenge. In recent years, different collaborative networking initiatives have emerged. Collaborative networks are of great value for science and technology institutions to share, generate, and disseminate new knowledge that can lead to innovations and form a solid basis for a national care network [22-24]. In this scenario, records represent an important tool in acquiring the necessary knowledge about the clinical form and natural history of patients with RD.

Maintaining records of epidemiological data also contributes to the planning of public health programs, which in some cases requires supranational coordination. Thus, European Reference Networks (ERNs) were organized, supported by a series of rules and guidelines that provide a cohesive structure for sharing good practices of diagnosis, treatment, and standardization of recommended approaches for RDs. The promotion of ERNs contributed to the identification of already established centers of expertise and encouraged the voluntary participation of health service professionals in ERNs dedicated to specific groups of RDs [22].

Italy was one of the first European countries to develop specific RD regulations. The success of the experience in Italy is exemplified by the regional network of Piedmont and Valle d'Aosta, where networked activities have provided several benefits, such as improvement of multidisciplinary knowledge, provision of quality care, and reduced cost of therapeutic mobility [22,23]. To produce epidemiological evidence on RDs and support health service policy and planning, Italy also assessed the integrity and consistency of procedures carried out from its national registry and found that the data quality still represents a limitation to any solid epidemiological estimate [22].

After comparing the outcome of patients with primary systemic amyloidosis in a referral center with the population of this same Italian network, another study in the country found that the patients observed by the network had a diagnosis 4 months earlier than those seen in the reference center. In addition to the rapid dissemination of knowledge pointed out as the main cause of this difference, important epidemiological differences were observed, which further reinforces the need for the standardization of reliable prognosis and the administration of clinical trial results [22,24].

According to the French National Plan for Rare Diseases, the first step in identifying patients with RD who are eligible for clinical trials or cohort studies is the definition of a minimum set of national data. In addition, providing reference centers with information technology (IT) tools contributes to the improvement of the service and research of RDs. Thus, according to international regulations on privacy and intellectual property and based on interoperability and semantics standards, the construction of the French model allowed data sharing in a national network composed of 131 centers specialized in RDs [22,24-26].

One of these French national reference centers went further and created a web-based medical archive of pediatric interstitial lung diseases. The construction of a national database made it possible to centralize and serve various stakeholders, such as researchers, clinicians, epidemiologists, and the pharmaceutical industry. Consequently, with the increasing engagement of new participants and the creation of committees to control data quality, there was an increase in the accuracy of the information provided, and several alternative solutions, depending on local possibilities, were configured [27]. Similar initiatives have also taken place in Germany [28] and the United Kingdom [29].

To not only collect records but also analyze them, a conceptual and digital framework based on the Asia-Pacific Economic Cooperation Rare Disease Action Plan has been articulated [26]. A proposal for a rare disease registry and analytical platform aims to assist in clinical decision making and improve the design and delivery of health services [30].

On the other side of the ocean, the National Center for the Advancement of Translational Sciences, one of the 27 departments of the US National Institutes of Health (NIH), maintains initiatives that aim to enhance the research of rare diseases, such as the promotion of information sharing and the construction of multidisciplinary collaborations. The Rare Diseases Clinical Research Network, for example, despite being formed by distinct clinical research consortia, shares the same data coordination and management center [31].

This management is only possible due to the availability of a genomic database maintained by the Genetic and Rare Diseases Information Center and the RD record program based on international standards and sharing (eg, Health Level Seven, Human Phenotype Ontology [HPO]) as well as the toolkit for the development of patient-focused therapies (National Center for Advancing Translational Sciences Toolkit), represented by an information portal with guidelines for the development process of research and partnerships with the NIH and the Food and Drug Administration [32].

Worldwide, these two actions integrated not only clinical and epidemiological data and records but also information from biorepositories of biological samples for rare biospecimens (RD-HUB) [33], and they created an integrated platform that connects databases, records, biobanks, and bioinformatics clinics for rare disease research (RD-Connect) [34]. To address the quality of these data, several models and tools have been developed worldwide [34,35]. An assessment approach for diagnosing rare diseases based on Unified Modeling Language and ontologies, called FindZebra, improves the quality of diagnosis compared with standard search tools [34-36].

To become more than a search engine, decision support systems for clinical diagnosis have incorporated artificial intelligence and natural language processing techniques to provide more accurate and useful systems [37]. By having the infrastructure established according to the Ministry of Health, we can focus on the data analysis. Data science has been playing a major role in retrieving insights from patient reports and human and technical resources. Thus, the main goals of this project are described in the following section.

### Objectives

The primary objective of this study is to identify the essential elements for mapping, infrastructure, and data analysis for the Brazilian Network of Rare Diseases. Secondary objectives are to (1) create and implement a system that allows the integration of data available in different systems of health care, social assistance, and epidemiology of RD cases (a shared electronic medical record), simplifying the access to patient data via the web by health care stakeholders; (2) promote interoperability between health information systems through the use of the Semantic Web combined with traditional communication and data exchange techniques for functional and semantic interoperability and the integration of databases to improve the management of health services data; (3) develop a single and complete database using cloud computing with an access hierarchy and well-defined security rules by building a ubiquitous platform capable of providing access services and adding syntactic and semantic value to data, covering innovative techniques such as the use of blockchain for cloud computing; and (4) develop an evidence-based portal with national protocols for monitoring and analyzing data collected or produced in several RD patient care settings, incorporating data processing, analysis, and machine learning techniques to assess the clinical situation and possible patient risks in real time.

## Methods

### Brazilian Rare Disease Network

Typically, information technology investigations can be distinguished as applied basic research. Basic research is scientific research focused on improving the understanding of phenomena and events [38]. Applied research uses scientific studies to develop technologies and methods to intervene in natural or other phenomena, aiming to improve human interaction with such phenomena [39].

As mentioned, the study described is part of a larger project, entitled the Brazilian Rare Disease Network, with a collection of quantitative data coupled with an innovation proposal, the creation of an epidemiological surveillance service network involving university hospitals, Reference Services for Neonatal Screening (Serviços de Referência em Triagem Neonatal [SRTNs]), and Reference Services for Rare Diseases (Serviços de Referência em Doenças Raras [SRDRs]) throughout the Brazilian territory.

Considering the goal of consolidating a national network of rare diseases that covers all regions of Brazil, this study has the participation of SRDRs, university hospitals that may or may not belong to the Brazilian Hospital Services Company (Empresa Brasileira de Serviços Hospitalares) network, and SRTNs. These centers are essential for building a national database that efficiently maps and represents the situation of the field of rare diseases in a country [40]. Brazil is divided into 5 regions (north, northeast, midwest, southeast, and south). The chosen participating centers are distributed across all Brazilian regions and are units of reference in health care for the population of their respective localities, according to the National Policy on Comprehensive Care of People with Rare Diseases [9].

Participating health centers are divided as follows by country regions: 6 centers in the north, 11 in the northeast, 6 in the midwest, 12 in the southeast, and 5 in the south. These include 16 Brazilian capitals that together have a total of 47 million people. In addition, as they are referral centers, they have the infrastructure to receive patients from smaller municipalities for the diagnosis and care of their population.

The area of care for people with rare diseases is structured into primary care and specialized care, following the Health Care Network (Rede de Atenção à Saúde) and the Guidelines for the Comprehensive Care for People with Rare Diseases plan of the SUS. SRDRs are responsible for preventive, diagnostic, and therapeutic actions for individuals with rare diseases or at risk of developing them, according to care axes. The SRDSs have a network of Specialized Rehabilitation Centers (Centros Especializados em Reabilitação [CERs]), which can receive patients referred from SRDSs and assist in the rehabilitation of these patients [41].

The CERs are structural components of the National Policy on Comprehensive Care of People with Rare Diseases. According to the integrality of care, these centers perform treatment, concession, adaptation, and maintenance of assistive technology, constituting a reference for the health care network in the territory [42]. SRDRs and CERs work together with university hospitals to promote comprehensive and universal care for rare disease patients.

The traditional concept defines a university hospital as an institution that is characterized by four traits: being an extension of a health teaching establishment (of a medical school, for example), providing university training in the health field, being officially recognized as a teaching hospital and subject to the supervision of competent authorities, and providing more complex medical care (tertiary level) to a portion of the population and being able to receive patients from SRTNs [42,43].

The SRTNs are units with multiprofessional health teams accredited and specialized in assistance, follow-up, treatment, and redirection of newborn patients diagnosed with pathologies such as phenylketonuria, congenital hypothyroidism, sickle cell diseases, biotinidase deficiency, congenital adrenal hyperplasia, and cystic fibrosis. Such pathologies are detected in the SRTN’s own or an outsourced laboratory, according to the rules established in the National Neonatal Screening Program [44].

Initially, the 3 main collaborator groups consist of 17 university hospitals, 6 SRTNs, and 17 SRDRs. The effective consolidation of the Brazilian network of rare diseases, based on the mapping of these services, depends on 3 steps: (1) approval by the ethics committee of the coordinating institution of the project, (2) approval of the local ethics committees of each participating institution, and (3) consolidation of the human resources participating in each institution through the institutional consent form.

The first step has already been completed and the others are in progress. Any divergence in these steps results in the exclusion of the participating center from the project. While these steps are in progress, representatives of all participating institutions meet monthly—on the second Saturday of the month in the morning—to discuss and structure the other activities of the project. Additionally, institutions must disseminate and invite partner services to participate in the initiative. The structuring and alignment of the final group of participants in the Brazilian network of rare diseases was finalized in August 2020.

### Ethical Considerations

The National Network of Rare Diseases project was approved (Edital No. 25/2019) from CNPq, with financial support from the Ministry of Health in the amount of R $3.5 million (US $662,139.10) [45]. Moreover, the main project was sent to the research ethics committee of Porto Alegre Clinical Hospital of the Federal University of Rio Grande do Sul (Hospital de Clínicas de Porto Alegre da Universidade Federal do Rio Grande do Sul) through Plataforma Brasil, a Brazilian platform of the Ministry of Health projects. The research ethics committee of Porto Alegre Clinical Hospital analyzed the research project (under code number 33970820.0.1001.5327 of Presentation Certificate for Ethical Appreciation). The research was approved (opinion number 4.225.579) on August 14, 2020.

To ensure the anonymity of patients while making it possible to track them if necessary, a password will be created for all patients, consisting of the first 2 letters of the city followed by the center number with 2 digits (from 01 for each city) and a 2-digit sequence for the patient's number. The rights, safety, and well-being of the subjects involved in the study will be the most important considerations and should prevail over the interests of science and society.

Considering the governmental efforts (ConecteSUS) [46,47], we similarly propose the use of a permissioned distributed blockchain solution that uses a key pair (private and public key) and a symmetrical consortium key for data encryption. A consortium distributed storage network will be established, consisting of research centers and other approved stakeholders throughout Brazil [48].

Authentication, authorization, integrity, and confidentiality verification mechanisms will be implemented through the establishment of a security layer. Thus, the security structure presented in this project aims to protect sensitive data for interoperability purposes. All computational techniques that support the solution, such as encryption and hashing, are well-known technologies that, when combined, can offer robust security features. In this way, each candidate system to interoperate with the rare disease ecosystem can easily meet all the necessary technical requirements.

All data collection processes will match the novel Brazilian General Law of Data Protection (federal law No. 13.709/18) [49]. The law refers to the respect to user privacy, transparency in the data collection, security, and prevention of damage in personal data. Since August 16, 2020, the law covers all national territory, and its violation can cause a warning, penalties, a data block, and suspension of the project [50]. As mentioned, the project will ensure the anonymity of the data during analysis. In addition, the IT team will present to all members of the network the definition and main aspects of the General Law on Protection of Personal Data (*Lei Geral de Proteção de Dados Pessoais*) using supporting materials and a patient consent form for data collection and usage, with full transparency.

### RARASnet Project Management

The project management will include the cooperation and execution of several activities, including technological and technical implementation, that must be harmonized. The technical IT group will coordinate activities related to electronic resources, such as data collection instruments design, database management, and data analysis. The IT team is also responsible for maintaining a communication channel with the project's principal investigators to receive clinical administrative and clinical research input.

A set of practices that merge development and operations (DevOps) will be used as a reference to standardize the development process and align activities of software engineering, infrastructure operation, and quality assurance. As an agile methodology, DevOps allows quick delivery of a small set of requirements from concept to deployment. The method also creates efficiency in results monitoring due to continuous integration and the appreciation of high-value feedback from all stakeholders [51].

For project management and to increase collaboration across team members, Trello (Atlassian) [52] will be used, which provides easy visualization of tasks and priorities, as well as a macrovision of development stages. The workflow of a data analysis project will follow the classic steps of a knowledge discovery in databases process [53], detailed in the following subsections.

### Data Collection Procedures

Initially, the instruments to be used in data collection will be framed, validated, and tested. These instruments should serve as a basis for the steps that involve the survey of retrospective data in the participating institutions and as a model for the stage involving the prospective survey and analysis. Based on an initial report characterizing the informational maturity of the collaborating institutions, online training will be given to address the functioning of the data collection instrument developed, validated, and tested for the project's retrospective phase, and the same process will be carried out later in the prospective phase of the project.

The collection will be carried out through access to medical records, with data recording on portable computers acquired with funds from this proposal and carried out by fellows of the project with the support of researchers from each service. Data quality indicators will be monitored in this intervention, mainly about the difficulties encountered by institutions to codify the diseases in an interoperable way, ensuring the production of a reliable picture of the maturity of data collection of rare diseases in Brazil.

To ensure the monitoring of data quality indicators, an early hearing detection and intervention (EHDI) will be conducted, and dimensions such as completeness, uniqueness, timeliness, validity, accuracy, and consistency will be evaluated [54]. Elements not present in the EHDI, such as acceptability, reliability, and flexibility, will also be considered; the use of dimensions will vary depending on the requirements of each center. These indicators were selected based on their importance in monitoring and evaluation in the National Policy on Comprehensive Care of People with Rare Diseases. It will also allow tracking results from the source to the national level and be indicative of data quality for all the indicators within a program area [55].

During data collection, phenotypic data will be described according to HPO terms, restricted to 5 terms per case, allowing the description of phenotypes of known syndromes. Information about the coding of the disease will also be presented, considering the name of the disease, the International Classification of Diseases 10th Revision (ICD-10), the Orpha number, and the gene name or symbol, thus allowing comparison with data from other platforms, such as Orphanet.

Data collection instruments (ie, case report forms [CRFs]) will be established by principal investigators and applied in distinct project phases, each with a specific objective. The development of all CRFs is guided by the National Policy of Comprehensive Care for People with Rare Diseases [56,57] in the context of the Brazilian Health Public System.

The main instruments are (1) a survey of the technical and technological resources of the participating research center, used to recognize needs and prepare and provide resources for data integration and collection across research centers; (2) a survey of procedures performed at participating centers, used to recognize the availability of technological resources for genetic diagnosis and human resources in the assistance of individuals with rare diseases; (3) a retrospective collection of clinical data, that is, the characterization of the clinical profile of patients with RDs treated throughout the country in the last 2 years; and (4) a prospective collection of clinical data, that is, the follow-up of patients with the defined RD clinical profile treated throughout the country, for the identification of changes in the clinical profile, such as in diagnosis and treatment.

After the initial development, the validation phase will take place. Key researchers, along with main investigators, will perform several rounds of revision and validation for each CRF. This process will occur until researchers reach a consensus. Then, the final version of an instrument (usually a paper-based one) will be translated into an electronic-based version.

### Computational Infrastructure and Data Collection Resources

The study will rely on a computational infrastructure to satisfy technological needs during all project phases. First, cloud computing resources were acquired as an infrastructure as a service. This makes it possible to quickly scale up and down with demand. Additionally, the expense and complexity of buying, managing, and maintaining physical servers and other data center infrastructure are avoided [58].

In this case, the University of São Paulo provides a private cloud computing environment (interNuvem USP) and manages the whole infrastructure, while the project's owners only need to install, configure, and manage their own software, operating systems, and applications. Several resources, such as web, database, and data collection servers, will be available to help deliver this project outcome.

During the project, it will be necessary to collect data using CRFs. To facilitate the creation of electronic CRFs and their distribution, REDCap (Research Electronic Data Capture) and KoBo Toolbox will be used as electronic data capture systems. REDCap was built in 2004 by a team at Vanderbilt University to enable classical and translational clinical research, basic science research, and general surveys, providing researchers with a tool for the design and development of electronic data capture tools [59].

KoBo Toolbox, developed by the Harvard Humanitarian Initiative, is a free and open-source suite of tools for field data collection and basic analysis. It was initially built for use in challenging environments in developing countries, but it can be extended to any type of research [60]. Both electronic data capture systems are free, although licensing is necessary for REDCap. After applying for a REDCap license of use, the RARAS REDCap Server was established, which is now part of the REDCap Consortium, a community of experts and REDCap administrators [61]. KoBo Toolbox does not demand a licensing process and the software is publicly available for download and installation.

REDCap and KoBo Toolbox are integrated and can be used together. The first is used for data research, data storage, reporting, analysis, and management. The second is used exclusively in the data collection process as a front-end tool for final users, allowing responsive and offline data collection on any type of device without the need to install any third party or additional mobile app. After submitting a record in KoBo Toolbox, data are instantly synchronized with the REDCap database. This integration is possible due to a framework developed by the IT group.

### Database Modeling

By exploring the data sources of the Orphanet platform related to information on medicines and rare diseases, we started the modeling phase of the database. Additionally, materials were selected for the knowledge acquisition phase for the development of a computational ontology that will reuse the Orphanet Rare Disease Ontology (ORDO) [62], thus helping the classification and hierarchization of bibliographic data on the prevalence of these diseases. After this initial analysis to select the best attributes (variables) that represent this health domain and are aligned with the profiles of the participating centers, the second stage of modeling the database is expected to start [25].

The first step is important so that the system does not request variables that are not relevant to the study, reducing the time taken to collect patient data by the health professional. More specifically, conceptual modelers describe structure models in the form of entities, relationships, and constraints, as they can also describe behavioral or functional models in terms of states, transitions between states, and actions performed on states and transitions. Finally, they can describe interactions and user interfaces in terms of messages sent and received and information exchanged. At the end of the first stage, a system requirements document must be prepared, detailing all functional and nonfunctional aspects of the implementation and application layers [31].

To facilitate the understanding of the information flow and operational processes of the participating institutions, auxiliary diagrams will be produced using the Business Process Management Notation approach. Such documents will be used during the project to validate the information from the services, which will also be useful for the implementation and maintenance phases of the database.

The second phase of the modeling, therefore, consists of mapping the model in the form of relational tables. To ensure data consistency, the mapping is done according to the rules of the relational model, which was chosen because of its simplicity and robustness and because it uses structured query language (SQL), which has become common in relational databases. To generate the first model of the proposed database, the MySQL Workbench (Oracle Corp) software will be used, which allows data management and SQL queries to be built and facilitates the administration, creation, and maintenance of several databases in the same location. In this way, the bank will be ready for use and its implementation will be dynamic, offering the scope for future updates and maintenance [32].

### Data Quality Assurance

As previous stated, both the retrospective and prospective phases will collect study data using the KoBo Toolbox electronic data capture tool and store them using the REDCap server hosted at the Ribeirão Preto Medical School, University of São Paulo, Brazil. The KoBo Toolbox online data entry system will minimize the data entry errors and facilitate the monitoring and quick resolution of queries and missing data.

The data collection tools will be reviewed by other researchers and pretested on a convenient sample of records and clinical settings. Reviewers will note their individual experience with both the definitional criteria and the time taken to collect and record data. Based on the final pretest, revisions will be made to both data collection instruments.

A manual of operations will be developed to minimize the need for judgment and interpretation by the data collectors and to increase the quality of data collection done by the health care center professionals. The manual of operations will include a description of the study in general terms, emphasize the importance of complete and accurate data, and foster the standardization of data collection.

The responsible health care center staff member will maintain a problem logbook to document unanticipated problems. Technical questions encountered in the field will be resolved through consultation with the technical team and researchers responsible for the project.

**Figure 1:**
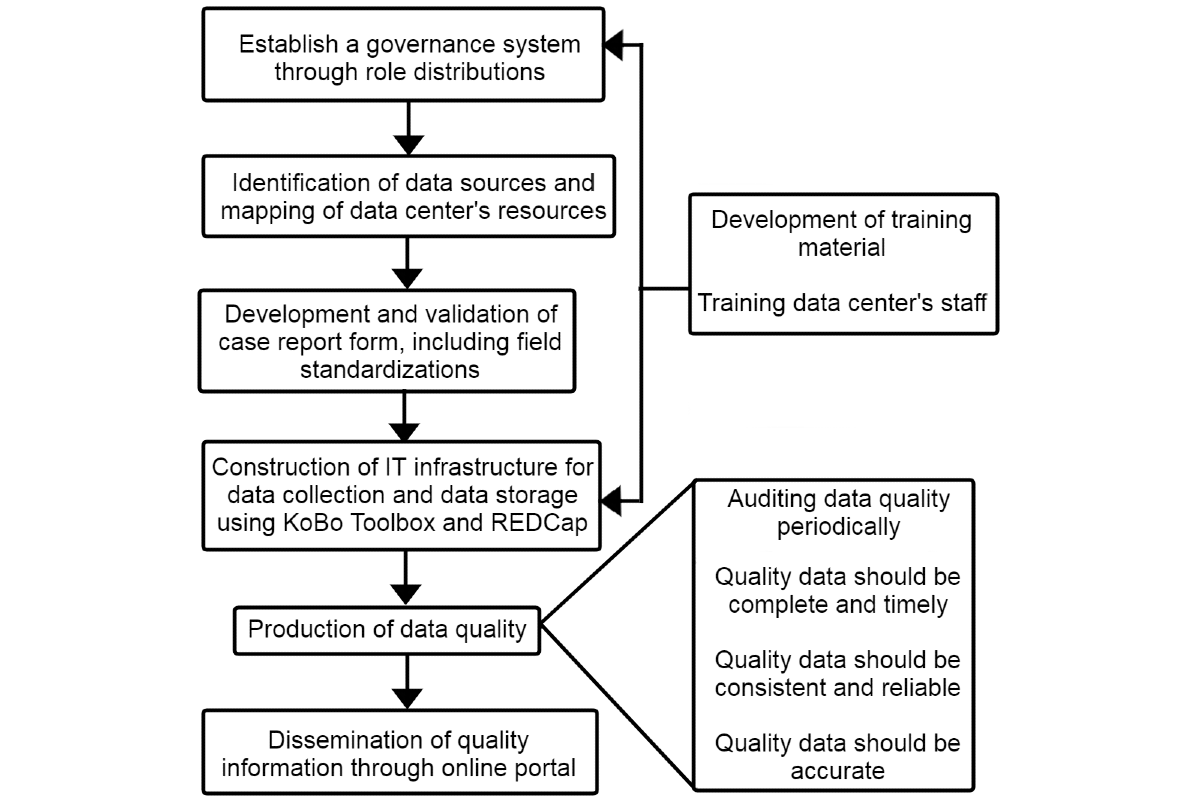
Framework for quality management of rare disease registries. IT: information technology.

To ensure that the record quality fulfills all prerequisites described in the literature and the normative documents previously mentioned, we will follow a set of recommendations described by the ERN in the RD-Connect framework, incorporating the indicators in each step of the process collection, storage, preprocessing, processing, and reporting [63,64]. Our plan will consider aspects such as governance standards, infrastructure in compliance with the FAIR principles (findable, accessible, interoperable, and reusable for humans and computers), didactic material, and informative documents, as well as personnel training and a data quality trail. The process and tools used for each level are presented in Figure 1.

### Data Management

Trained research nurses at the participating health facilities will use KoBo Toolbox data collection tools to collect data for both retrospective and prospective phases. All entries will be deidentified at the stage of data collection, and participants will be identifiable only by unique identification codes that are only accessible and known to the hospital coordinator. A customized data entry and monitoring system will be developed in the REDCap platform for this study. This data entry system will be password protected and accessible only to the database managers and study team. The system will be developed and coordinated by the study data management unit at the University of São Paulo, Brazil.

### Portal Development and Data Analysis

After the identification and collection of the essential data, the IT team will be responsible for developing all the data analysis by the supervisors of specialists in the rare disease network. The analysis will serve as support for RARASnet specialists and patients to understand the main aspects of human and technical resources and the flow of rare diseases in Brazil. As retrospective and prospective data will be collected, they will serve as a base for the exploration of statistical and modeling computational methods, and with the validation of the results among specialists, the database will be incorporated into DATASUS and communicated in scientific reports and a web portal. This web portal will be one of the main practical contributions of this work. It refers to the Brazilian Digital Atlas of Rare Diseases, available through a health observatory, which aims to integrate structural information about the referential institutions working in rare diseases in the country and clinical information about the individuals assisted by these institutions. This building process will be done according to the guidelines proposed by WHO for the development of health observatories [65].

From that data organization, the analysis tools will be made available, providing health indicators to the managers (hospital, municipal, and regional). The main analyses of the web portal will be (1) the flow of patients, which will present the displacement of patients according to the place of origin and the hospital care through georeferenced maps and tables; (2) hospital indicators, which will provide the automated calculation of 31 hospital indicators, such as mortality, morbidity, capacity, and usability, aiming to observe and compare these indicators among institutions; (3) nosological profiles, which will highlight the hospital care of individuals, allowing for the characterization of morbidities in the rare disease community; (4) diseases sensitive to primary care, which will describe hospitalizations for morbidities related to primary care, facilitating the identification of hospitalization rates that could be avoided by strengthening primary care; (5) prediction of risk of death by the Charlson Comorbidity Index, which will provide the risk of death for patients according to their comorbidities; and (6) medical procedures, which will describe the surgical procedures performed, allowing the comparison between these procedures and the resources used [66-69].

The tools described will provide interactivity through consultation filters with spatial disaggregation (by region, health region, municipality, or a specific hospital) and temporal disaggregation. Thus, information will be able to be explored historically, geographically, and in real time, supporting different demands and decision making for rare diseases in Brazil. However, besides the web portal, which will contain general public information and resources, we will also provide all the knowledge through videos and talks using didactic language to facilitate understanding.

## Results

Considering the objective list and proposed conceptual and technical model, RARASnet presents some outcomes of interest, both specific and collaborative, in 9 steps:

1. Survey epidemiology, clinical procedures, and therapeutic resources, such as the number of individuals with rare disease according to each diagnostic group, age, race, sex, and other features.
2. Create a national rare disease network with the participation of important university hospital health services in rare diseases to create a database of national rare diseases.
3. Cover all regions of Brazil concerning the main rare diseases, with institutes and number of cases stratified according to each type of condition.
4. Create a standard in sociodemographic, epidemiological, clinical, and therapeutics data with the advice of the specialists in the national rare disease network. The data should follow patterns proposed by the Ministry of Health guidelines and HPO terms.
5. Identify the type of treatments being applied in each center and those funded by SUS or by supplementary health. The goal is to have a quantitative analysis for each type of treatment to understand the overall status of rare disease in Brazil and perform public health policies.
6. Map existing diagnostic and technological resources within the network.
7. Map human resources, such as the quantity of workers and specialists available in the network in each region of Brazil.
8. Establish a network of partners to underpin collaborative studies concerning rare diseases.
9. Develop the online Brazilian Atlas of Rare Diseases according to the guidelines of the WHO [70] to help professionals, the general public, and political decisions.

**Figure 2:**
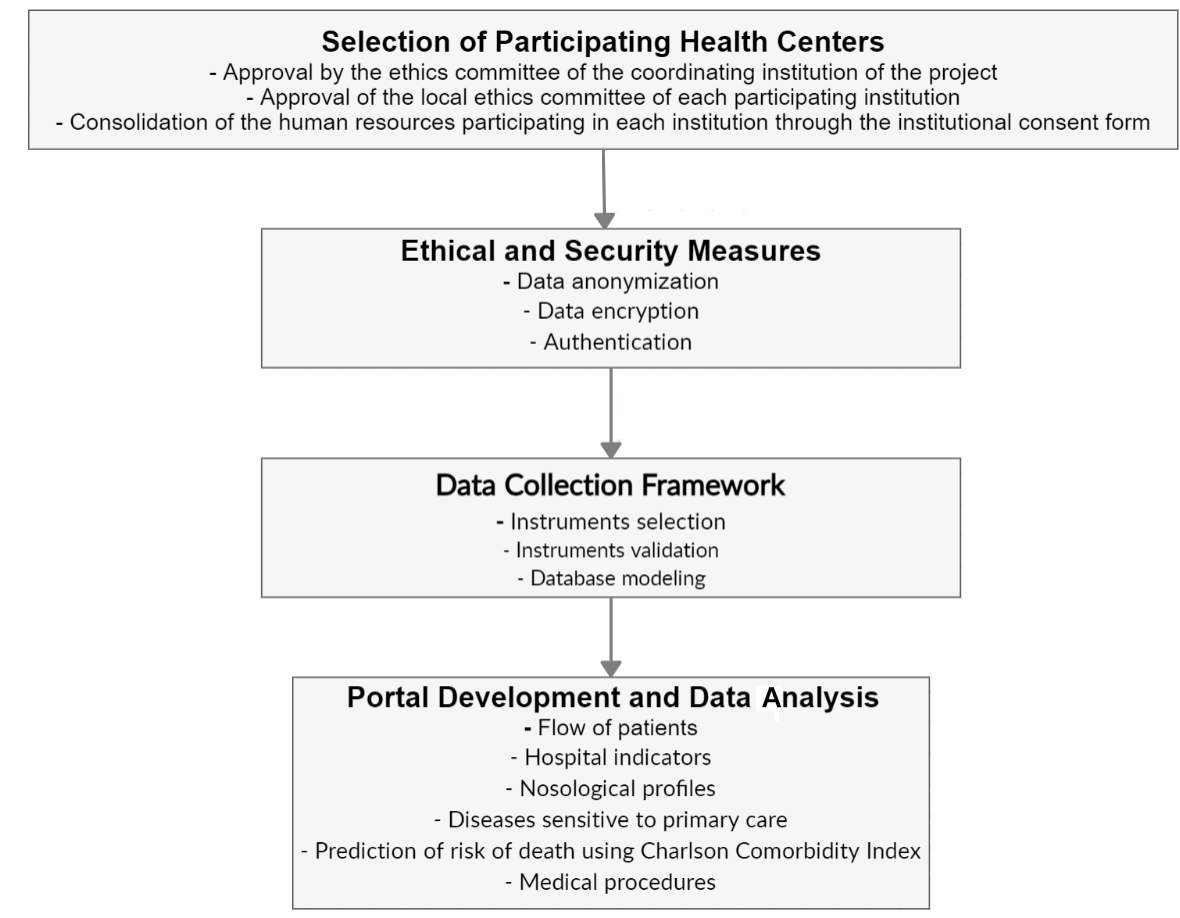
Main activities of RARASnet.

The present project is in its initial stages, and a survey was completed by each reference center to evaluate the technical aspects of each health care center, such as the presence of computers, technical support staff, and a digitized system. Moreover, we are in the process of internally validating the collection instruments with specialists and principal investigators and preparing the pilot project to be carried out at the coordinating center for external validation. All the predicted methodological processes are shown in Figure 2.

For the participating centers that have already obtained the project approval from their respective ethics committees, we developed an initial data collection instrument to verify the technological infrastructure of each center and the way these institutions capture information related to rare diseases. This survey aims to list and categorize these institutions according to their methods of data storage and retrieval, which can be digital, through electronic medical records and management software, or analog, through paper-based record management.

From a total of 40 participating centers so far, 39 have already responded to this initial survey, and among these, the results showed that 23 institutions (59%) have an electronic collection and recovery system and 16 institutions (41%) have a paper collection system. This initial survey is important for our IT team to plan the best clinical data collection approach for each institution during the project, aiming to minimize obstacles through an adequate and personalized collection proposal for each center.

One of the biggest challenges of this study is aligning data collection in all participating institutions so that the process of recovering data from medical records is standardized regardless of the storage support and the methods of retrieving information used in each health unit. In our scenario, based on 39 institutions, 14 (36%) health units extract information from medical records exclusively on paper, while 2 (5.1%) have a nonapplicable data recovery method; although they store their data in a system or on paper records, their recovery process does not fit into one of these methods (eg, using applications that are not for this purpose).

Later, we will standardize and analyze the clinical and epidemiological data and use these data to develop the national network for monitoring rare diseases, using the Digital Health Observatory to make the information available.

The project had its financing approved in December 2019. Retrospective data collection started in October 2020, and we expect to finish in January 2021. We will begin the prospective data collection in February 2021, and we expect to finish in June 2021. During the third quarter of 2020, we enrolled 40 health institutions from all regions of Brazil. We are currently receiving data to be analyzed. We expect the publication and dissemination of the findings in the second half of 2021.

## Discussion

This study is currently in its initial stage. We have performed a survey of technical aspects of health care centers (eg, support staff and technological infrastructure). A pilot data collection of clinical data carried out by specialists and principal investigators is planned to underpin the instruments’ validation.

### Main Problems Anticipated and Proposed Solutions

The heterogeneity of the data is intrinsically connected to the type of information generated by the health services, which is considered diverse and complex. Some of the main problems normally encountered when handling health data are the highly heterogeneous and sometimes ambiguous nature of medical language and its constant evolution; the huge amount of data generated constantly by the automation of hospital processes; the emergence of new technologies; the need to process, analyze, and make decisions based on this information; and the need to ensure the safety of data related to patients [63].

To mitigate problems of heterogeneity and data standardization, we will make use of some semantic web technologies, which are presented as a fundamental approach to guarantee semantic interoperability and the integration of dispersed and isolated data sets. More specifically, we will use biomedical ontologies, which provide controlled vocabularies of scientific terminologies used to assist in the annotation of produced data, such as basic terms and their relations in a domain of interest, as well as rules to combine these terms and relations [64]. As mentioned, some of the ontologies used will be ORDO and HPO.

Once the ontologies are defined, it will be possible to perform the semantic markup on the collected records present in our relational database and provide a SPARQL Protocol and RDF Query Language access point to execute queries on the data set, allowing us to make available a set of data that can be extracted by different information systems, as long as they are connected to the web.

For security reasons related to the sensitivity of the stored information, direct access by external systems to the data structure is blocked by default. Therefore, an authorization layer will be built to support the authentication processes (validation of the identity of external systems). The authorization and protection of the information transmitted will use digital signature and hybrid encryption techniques, that is, a combination symmetric (unique key) and asymmetric (public and private key pair) encryption.

We believe that through these planned solutions, obtaining information from the set of data related to rare diseases in Brazil will become possible, allowing data to be shared, reused, analyzed, and applied in other information systems, either to improve the completeness of other bases or to produce relevant knowledge to support decision-making processes in the context of rare diseases.

### Applicability of the Results

In the development of digital products and services for this project, all tools must ensure that users have the freedom to interactively navigate and filter data to visualize the analysis according to personal interests. For this, the Brazilian Digital Atlas of Rare Diseases will have a filter that allows spatial disaggregation (queries by regions, health regions, municipalities, or a particular hospital) and temporal data. The filters will allow the user to set different visualization schemes without accessing the raw data and modifying the database.

It will also be possible to perform other types of data aggregation in queries, such as grouping by gender, age group, ICD-10, Orphacode, Online Mendelian Inheritance in Man (OMIM) [63], phenotypic characteristics, and other information that the health professionals involved consider relevant. It is important to emphasize that in the RD context, ICD-10 and OMIM are not able to cover all diseases with a unique identifier [71,72]. Thus, when ICD-10 is used as a filter, a further option box will be opened to distinguish between diseases with the same code. For OMIM, only genetic disorders are covered, and a note will be displayed on the website [63]. Orphacode [62], on the other hand, is the nomenclature that fits all RD diseases with a unique code due to its polyhierarchical nature [71].

This approach makes it possible to measure the performance of both the institution providing the health service and the care team. The analysis of efficiency and performance will be presented through dashboards and reports in real time, which can be used for the elaboration of new models based on the results.

The database of patients with rare diseases will allow an interactive epidemiological map and detail the care journey of the main rare diseases in Brazil. In this sense, it is expected that these developments can assist the evidence-based decision-making process for rare disease services in Brazil, bringing benefits to patients, health professionals, and managers.

### Plans for Validation, Dissemination, and Use of Project Results

The dissemination of the results will include the production of scientific papers in periodicals relevant to the area and the realization of scientific dissemination to the direct target audience and collaborators through workshops and training to the participating centers. Aiming for project integration and sustainability, we will make the ontologies developed available in the international repository of biomedical ontologies, BioPortal. These artifacts will therefore be able to be used in other projects around the world and updated constantly. BioPortal is an open database that provides access to biomedical ontologies via web services, facilitating the participation of the scientific community in the evaluation and evolution of ontologies by suggesting additional resources for mapping terminologies and reviewing criteria and standards [73].

With the main results and interest topics, we intend to recruit a multidisciplinary panel for an e-Delphi [74] consensus-building exercise with the ad hoc team members. The e-Delphi method is an interactive structured communication technique to reach consensus on the responses, and it comprises an initial open round of questions to revise or suggest a list of potential items for scoring in the subsequent two scoring rounds.

Once results are validated, it is crucial “to design strategies and solutions to overcome bottlenecks that prevent proven and innovative public health interventions” from reaching the people who need them [75]. For this purpose, we intend to use the WHO toolkit for implementation research. One of the WHO toolkit topics describes how to plan a rigorous research project, including identifying implementation research outcomes, evaluating effectiveness, and making plans to scale up implementation in real-life settings [76].

Once we have the findings, we intend to analyze the implementation of these interventions and strategies. For this, the reach, effectiveness, adoption, implementation, and maintenance (RE-AIM) framework [77] will be used to organize reviews of the existing literature on health promotion and disease management in different settings. RE-AIM is a tool used to translate research into action for digital technologies by measuring 5 essential dimensions for successful implementation: reach, effectiveness, adoption, implementation, and maintenance.

The overall goal of the RE-AIM framework is to encourage program planners, evaluators, readers of journal articles, donors, and policy makers to pay more attention to essential program elements, including external validity, which can improve the sustainable adoption and implementation of effective, generalizable, evidence-based interventions [78]. Finally, by applying the RE-AIM framework, we can emphasize responses to improve the chances that recommendations will have a positive and sustainable impact on public health.

## Abbreviations

CER: Specialized Rehabilitation Center
CNPq: Brazilian Council for Scientific and Technological Development
CRF: case report forms
DevOps: development and operations
EHDI: early hearing detection and intervention
ERN: European Reference Network
HPO: Human Phenotype Ontology
ICD: International Classification of Diseases
IT: information technology
NIH: National Institutes of Health
OMIM: Online Mendelian Inheritance in Man
ORDO: Orphanet Rare Disease Ontology
RD: rare disease
RE-AIM: reach, effectiveness, adoption, implementation, and maintenance
REDCap: Research Electronic Data Capture
SQL: structured query language
SRDR: Reference Services in Rare Diseases
SRTN: Reference Services for Neonatal Screening
SUS: Unified Health System
WHO: World Health Organization
